# An active Mitochondrial Complex II Present in Mature Seeds Contains an Embryo-Specific Iron–Sulfur Subunit Regulated by ABA and bZIP53 and Is Involved in Germination and Seedling Establishment

**DOI:** 10.3389/fpls.2017.00277

**Published:** 2017-02-28

**Authors:** Franko Restovic, Roberto Espinoza-Corral, Isabel Gómez, Jesús Vicente-Carbajosa, Xavier Jordana

**Affiliations:** ^1^Departamento de Genética Molecular y Microbiología, Facultad de Ciencias Biológicas, Pontificia Universidad Católica de ChileSantiago, Chile; ^2^Centro de Biotecnología y Genómica de Plantas – UPM-INIA, Campus de Montegancedo, Universidad Politécnica de MadridMadrid, Spain

**Keywords:** mitochondrial respiratory complex, succinate dehydrogenase, iron–sulfur subunit, abscisic acid, ABI3, bZIPs, *Arabidopsis* seed germination, seedling establishment

## Abstract

Complex II (succinate dehydrogenase) is an essential mitochondrial enzyme involved in both the tricarboxylic acid cycle and the respiratory chain. In *Arabidopsis thaliana*, its iron–sulfur subunit (SDH2) is encoded by three genes, one of them (*SDH2.3*) being specifically expressed during seed maturation in the embryo. Here we show that seed *SDH2.3* expression is regulated by abscisic acid (ABA) and we define the promoter region (-114 to +49) possessing all the *cis*-elements necessary and sufficient for high expression in seeds. This region includes between -114 and -32 three ABRE (ABA-responsive) elements and one RY-enhancer like element, and we demonstrate that these elements, although necessary, are not sufficient for seed expression, our results supporting a role for the region encoding the 5’ untranslated region (+1 to +49). The *SDH2.3* promoter is activated in leaf protoplasts by heterodimers between the basic leucine zipper transcription factors bZIP53 (group S1) and bZIP10 (group C) acting through the ABRE elements, and by the B3 domain transcription factor ABA insensitive 3 (ABI3). The *in vivo* role of bZIP53 is further supported by decreased *SDH2.3* expression in a knockdown *bzip53* mutant. By using the protein synthesis inhibitor cycloheximide and *sdh2* mutants we have been able to conclusively show that complex II is already present in mature embryos before imbibition, and contains mainly SDH2.3 as iron–sulfur subunit. This complex plays a role during seed germination *sensu-stricto* since we have previously shown that seeds lacking SDH2.3 show retarded germination and now we demonstrate that low concentrations of thenoyltrifluoroacetone, a complex II inhibitor, also delay germination. Furthermore, complex II inhibitors completely block hypocotyl elongation in the dark and seedling establishment in the light, highlighting an essential role of complex II in the acquisition of photosynthetic competence and the transition from heterotrophy to autotrophy.

## Introduction

Mitochondrial Complex II or SDH (succinate:ubiquinone oxidoreductase, EC 1.3.5.1) plays a central role in mitochondria as the only enzyme of two fundamental metabolic pathways: the TCA cycle and the respiratory chain. This complex associated to the inner mitochondrial membrane catalyzes the transfer of electrons from succinate to ubiquinone, generating fumarate and ubiquinol. Complex II is the simplest of the ETC complexes, and in most organisms, it contains four subunits (Yankovskaya et al., 2003; Sun et al., 2005). The flavoprotein (SDH1) contains the succinate binding and oxidation site, and interacts with the iron–sulfur protein (SDH2), which contains three non-heme iron–sulfur centers mediating the transfer of electrons to the membrane. The peripheral (matrix side) SDH1-SDH2 subcomplex is anchored to the membrane by two small integral membrane proteins (SDH3 and SDH4), which contain the ubiquinone binding and reduction site (Yankovskaya et al., 2003; Sun et al., 2005). Interestingly, additional subunits of unknown function have been described for plant Complex II (Millar et al., 2004; Huang and Millar, 2013).

Complex II subunits are all nuclear-encoded in *Arabidopsis thaliana* (Figueroa et al., 2001, 2002; Millar et al., 2004). Surprisingly, several of the complex II subunits are encoded by more than one gene in *Arabidopsis*. For instance, we have reported that two genes, named *SDH1.1* and *SDH1.2*, encode the flavoprotein and that SDH1.1 is the main flavoprotein subunit found in Complex II and is essential for gametophyte development (León et al., 2007). Three genes, designated *SDH2.1* (At3g27380), *SDH2.2* (At5g40650), and *SDH2.3* (At5g65165), encode the iron–sulfur subunit. Considering that in most organisms there is a single *SDH2* gene, the presence of three genes in *Arabidopsis* raises interesting questions about their roles during plant development. The three SDH2 proteins would be functional, since they are highly conserved when compared with their homologues in other organisms and contain the cysteine motifs involved in binding the three iron–sulfur clusters essential for electron transport (Figueroa et al., 2001). *SDH2.1* and *SDH2.2* genes likely arose via a relatively recent duplication event and are redundant. Indeed, both genes have similar exon-intron structures, encode nearly identical proteins and are similarly expressed in all organs from adult plants (Figueroa et al., 2001; Elorza et al., 2004). Moreover, the knockouts of *SDH2.1* and *SDH2.2* do not have any phenotype, and we have been unable to obtain double *sdh2.1/sdh2.2* homozygous mutants (Elorza et al., 2004 and unpublished results). In contrast, *SDH2.3* exon-intron structure is completely different from that of *SDH2.1* and *SDH2.2*, the encoded protein is only 67 % similar to SDH2.1 and SDH2.2, and *SDH2.3* is specifically expressed in the embryo during seed maturation. Indeed, Elorza et al. (2006) showed that *SDH2.3* mRNA begins to accumulate in maturing embryos, is abundant in dry seeds and declines during germination and early post-germinative growth.

*SDH2.3* highly specific expression during embryo maturation raises interesting questions about the regulatory mechanism. Using promoter fusions to the GUS reporter gene, we first showed that *SDH2.3* expression is transcriptionally regulated (Elorza et al., 2006). Then, using mutated promoters, we demonstrated that three ABRE (**ab**scisic acid **r**esponsive) **e**lements and a RY-like enhancer element are necessary for its embryo-specific transcriptional regulation (Roschzttardtz et al., 2009). ABRE and RY elements have been implicated in the seed-specific expression of SSP genes and late embryogenesis abundant proteins (LEAs) genes (Parcy et al., 1994; Busk and Pagès, 1998; Nambara and Marion-Poll, 2003). Furthermore, three master regulators of seed maturation belonging to the B3 domain transcription factors family, ABSCISIC ACID INSENSITIVE 3 (ABI3), FUSCA3 (FUS3), and LEAFY COTYLEDON 2 (LEC2) (Santos-Mendoza et al., 2008), control *SDH2.3* expression *in planta* (Roschzttardtz et al., 2009). In contrast, although ABRE elements are known targets for transcription factors of the basic leucine zipper (bZIP) family, the *in vivo* role of bZIP transcription factors in *SDH2.3* regulation was not assessed. Here we show that bZIP53 controls *SDH2.3* expression *in planta* and that bZIP53/bZIP10 heterodimers are able to activate the *SDH2.3* promoter. Furthermore, we demonstrated that ABA controls seed *SDH2.3* expression.

*SDH2.1* and *SDH2.2* are expressed at very low levels during seed maturation and in mature seeds and their expression is induced during germination and early post-germinative growth (Elorza et al., 2006; Roschzttardtz et al., 2009). Thus, data suggest that a SDH2.3 containing Complex II may have a role at these early developmental steps, and that SDH2.3 is gradually exchanged for SDH2.1/2.2 as the iron–sulfur subunit. Consistently, here we show using single *sdh2.1* and *sdh2.3* mutants, and double *sdh2.1/sdh2.3* mutants, that a Complex II containing mainly the iron–sulfur subunit SDH2.3 is already present in mature dry seeds, before imbibition, and that this Complex II with SDH2.3 has an important but not essential role at early stages of plant development. In contrast, Complex II is essential for chloroplast development and seedling establishment.

## Materials and Methods

### Plant Material and Growth Conditions

All *A. thaliana* plant materials used were in the Columbia (Col-0) background. Seeds of the T-DNA insertion mutants *bzip10* (SALK_106031, NASC ID: N606031), *bzip25* (SALK_119931, NASC ID: N619931) and *bzip53* (SALK_069883, NASC ID: N569883) were obtained from the Nottingham Arabidopsis Stock Centre, and homozygous mutants have been previously identified (Weltmeier et al., 2006). *bzip53* is a knockdown line with the T-DNA inserted upstream of the ATG initiation codon, *bzip10* and *bzip25* are knockout lines. Mutants in ABA biosynthesis were also obtained from the Nottingham Arabidopsis Stock Centre: *aba2-1* and *aba2-3* have impaired xanthoxin oxidation, *aba3-1* and *aba3-2* have impaired abscisic aldehyde oxidation (Schwartz et al., 1997; Barrero et al., 2006).

Two homozygous *sdh2.3* insertional mutants, called dSpm (*sdh2.3-1*) and DsLox (*sdh2.3-2*), and two homozygous *sdh2.1* insertional mutants, called GARLIC (*sdh2.1-1*) and SALK (*sdh2.1-2*), have been previously characterized (Elorza et al., 2004; Roschzttardtz et al., 2009). The *sdh2.3-*1 mutant was crossed with either the *sdh2.1-1* mutant or the *sdh2.1-2* mutant. F1 seeds from the *sdh2.3-1*/*sdh2.1-2* cross were germinated on Basta^®^ and kanamycin, and F1 seeds from the *sdh2.3-1*/*sdh2.1-1* cross were germinated on Basta^®^. To isolate homozygous double mutants in next generations, genotyping was performed as described (Elorza et al., 2004; Roschzttardtz et al., 2009).

Seeds were surface-sterilized, stratified for 48 h at 4°C in the dark and then sown on one-half-concentrated MS medium with 0.8% (w/v) agar. After 2 weeks at 22°C under long-day conditions (16-h/8-h day/night cycle), seedlings were transferred to soil and grown either under long-day conditions or short-day conditions (8-h-light/16 h-dark cycle).

To measure hypocotyl growth in the dark and assay germination, seeds were surface-sterilized, sown on half-concentrated MS agar, and stratified for 48 h at 4°C. TTFA, carboxin, malonate, or sodium azide were added as indicated in the Figures. For hypocotyl measurements plates were placed vertically in the dark for 6 days, at 22°C. After scanning in an Epson Perfection v700 Photoscanner, images were analyzed with Image J software^1^. For germination assays, plates were incubated horizontally at 22°C under long-day conditions, and germination was scored at different times based on radicle protrusion. Seedling establishment was evaluated at 10 days based on the emergence of true leaves.

### Constructs for Transformation of *Arabidopsis* Plants

A construct containing the *SDH2.3* promoter and 5′ UTR fused to the GUS coding sequence (-223/+49, numbers in relation to the *SDH2.3* transcription initiation site) has been previously described (P3 construct in Elorza et al., 2004). To shorten the promoter, forward primer sdh2.3F and reverse primer sdh2.3R (see Supplementary Table S1 for oligonucleotides) were used to amplify by PCR the region between -114 and +49, using *Arabidopsis* genomic DNA as template. Putative AuxRE and DOF elements were mutagenized by PCR, using the P3 construct as template. Two PCRs were carried for each mutant with the same template. For the promoter mutated in the AuxRE element (mAuxRE), one amplification was done with primers sdh2.3F and mAuxRE-R and the other was performed with primers sdh2.3R and mAuxRE-F. A mixture of both amplification products was used as a template for a third PCR with primers sdh2.3F and sdh2.3R. The same procedure was employed to obtain constructs mutated in DOF (mDOF) or in both AuxRE and DOF elements (mAuxRE/mDOF), using sdh2.3F, sdh2.3R, and the following “mutated” primers: for mDOF, forward primer mDOF-F and reverse primer mDOF-R, and for the double mutant forward primer mAUXRE/mDOF-F and reverse primer mAUXRE/mDOF-R. PCR products (-114/+49 wild type and mutated constructs) were cloned into pGEM-T plasmid (Promega), and the DNA fragments obtained by digestion with *Bam*HI and *Nco*I were ligated into pCAMBIA1381^2^.

Three PCRs were performed to fuse the -114 to -32 region of the *SDH2.3* promoter to the minimal cauliflower mosaic virus (CaMV) 35S promoter. First, we amplified the -114/-32 fragment using the P3 construct as template, forward primer sdh2.3F’ and reverse primer RYR. Then, we amplified the CaMV35S minimal promoter (-64 to +41) using forward primer sdh2.3/35S, reverse primer GUS35S-R, and pBI121 (Clontech) as template. Finally, we used as template a mixture of both amplification products for a third PCR with primers sdh2.3F’ and GUS35S-R. The same procedure was followed to fuse the-223 to-32 region of the *SDH2.3* promoter to the CaMV35S minimal promoter, using forward primer sdh2.3F2 in first and third PCRs. PCR products were cloned into pGEM-T, recombinant plasmids were digested with *Eco*RI and *Nco*I and inserts were ligated into pCAMBIA1381.

To fuse the fragment upstream of the *SDH2.3* transcription initiation site (-114 to -1) to the CaMV35S 5′UTR, four serial PCRs were carried out. In the four PCRs the same forward primer (sdh2.3F′) was used, in combination with different partially overlapping reverse primers designed to reconstitute the 5′UTR: 35S-5UTR1-R, 35S-5UTR2-R, 35S-5UTR3-R, and finally primer GUS35S-R. Final PCR product was introduced into pCAMBIA1381 as described.

Structures of all constructs were verified by DNA sequencing. *Agrobacterium tumefaciens* GV3101 was transformed by electroporation, and *Arabidopsis* plants (Col-0) by the floral dip protocol (Clough and Bent, 1998). Seeds of the T1 generation were selected for resistance to hygromycin. Soluble extracts of T2 seeds were assayed for GUS activity exactly as described (Roschzttardtz et al., 2009). Protein concentrations were determined (Bradford, 1976), and GUS activities expressed as nanomoles of 4-methylumbelliferone per hour per milligram of protein.

### Transient Expression Assays

The protocol of Yoo et al. (2007) was followed to isolate and transform protoplasts from fully expanded leaves of 6–8 week-old healthy plants grown under short-day conditions. Three types of plasmid DNA were used for protoplast transformation: a reporter plasmid with the wild type or mutated *SDH2.3* promoter (-114 to +49) fused to GUS in the pBT10-GUS vector (Sprenger-Haussels and Weisshaar, 2000), effector plasmids expressing bZIP or B3 domain transcription factors under the control of the CaMV35S promoter in the pUCpSS vector (kindly gift of Dr. Patricio Arce) and a plasmid expressing NAN gene under the control of the CaMV35S promoter (transfection efficiency control for normalization, Kirby and Kavanagh, 2002). For each transfection, 9 μg of reporter plasmid, 6 μg of each effector plasmid and 3 μg of the NAN plasmid were used. The overall amount of DNA was set to 30 μg by adding pUCpSS plasmid and used to transfect 3 × 10^4^ protoplasts in 200 μl. All transfections were done in triplicate. Protoplast lysates were prepared in GUS buffer (Jefferson, 1987), and GUS and NAN enzyme assays were performed as described by Kirby and Kavanagh (2002).

To generate the reporter plasmid, the *SDH2.3* promoter region was amplified by PCR using *Arabidopsis* genomic DNA as template, forward primer sdh2.3F3 and reverse primer sdh2.3R. Mutated *SDH2.3* promoter was obtained by using as template genomic DNA from transgenic plants carrying the *SDH2.3* promoter mutated in the ABRE2 element (Roschzttardtz et al., 2009). PCR products were cloned into pGEM-T plasmid, and DNA fragments obtained by digestion with *Hind*III and *Nco*I were ligated into pBT10-GUS digested with the same restriction enzymes.

Effector plasmids for the expression of *Arabidopsis* bZIP10, bZIP25, bZIP53, and ABI3 were generated by cloning the corresponding cDNAs between the CaMV35S promoter and terminator in pUCpSS. cDNAs were obtained by RT-PCR with the following primer pairs: bzip10F and bZIP10R, bzip25F and bzip25R, bzip53F and bzip53R, and abi3F and abi3R (Supplementary Table S1). PCR products were cloned into pGEM-T, recombinant plasmids were digested with the appropriate restriction enzymes and the DNA inserts were ligated into pUCpSS.

Structures of all constructs were verified by DNA sequencing and plasmids DNAs were purified by centrifugation in CsCl gradients.

### Expression Analysis by Real-Time qRT-PCR

The method of Oñate-Sanchez and Vicente-Carbajosa (2008) was employed to extract total RNA from 25 mg of seeds. cDNA synthesis was carried out with 2 μg of DNase I-pretreated RNA, M-MLV Reverse Transcriptase (Promega), and random hexamers as primers. To evaluate gene expression, the primer pairs were RTsdh2.3F and RTsdh2.3R for *SDH2.3*, and RTclnF and RTclnR for clathrin. One tenth of the cDNA was employed in qRT-PCR assays, using the Mx3000P QPCR System (Stratagene) and the SensiMix Plus SYBR kit provided by Quantace. Expression values were calculated considering the amplification efficiency of each primer pair, and were normalized using the clathrin transcript as an internal control.

### Succinate Dehydrogenase Activity Staining

A simplified procedure was used, based on that described by Baud and Graham (2006). All steps, from imbibition to staining, were performed in the absence or presence of 1 or 10 μM cycloheximide. Seeds were imbibed in the dark for 16 h at 4°C, embryos were excised, incubated for 2 h at 37°C in 50 mM sodium phosphate (pH 7.6), and finally stained by incubation for 1–3 h at 30°C in 50 mM sodium phosphate (pH 7.6), 7.5 mM succinate (freshly prepared) and 2.4 mM nitroblue tetrazolium. In parallel, embryos were incubated in the same buffer without succinate, and show negligible background activity (**Figure 4**). After staining, embryos were washed with 70% ethanol, viewed in a stereomicroscope (Nikon SMZ800) and photographed with a Nikon Coolpix 4500CCD camera.

### Statistical Analyses

Krustall–Wallis test followed by Dunn’s test for multiple comparisons, and Mann–Whitney test were performed using GraphPad Prism, GraphPad Software (San Diego, CA, USA). Student’s *t*-tests were performed using the algorithm embedded into Microsoft Excel.

## Results

### *SDH2.3* Promoter Characterization

**FIGURE 1:**
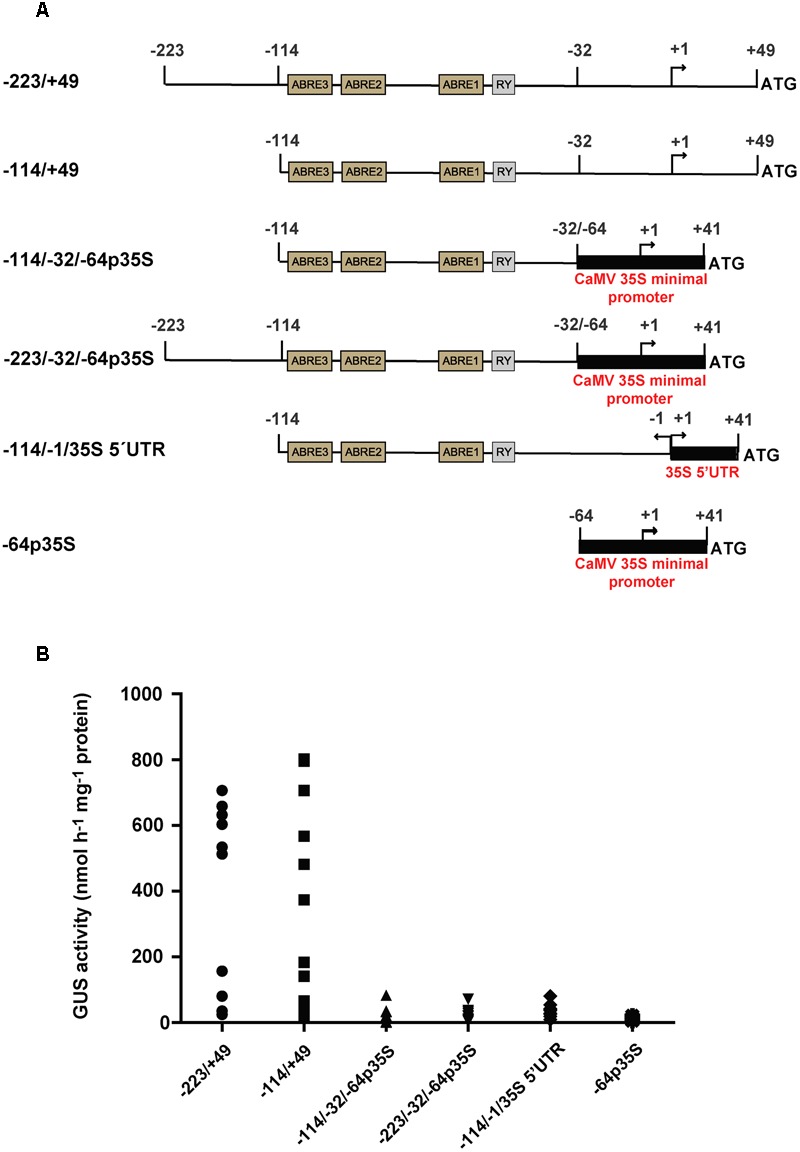
**Identification of the *SDH2.3* functional promoter. (A)** Structure of the constructs fused to GUS. Thin lines with motifs shown in boxes represent the *SDH2.3* promoter. Numbers are in relation to the transcription initiation site, indicated by a curved arrow. Black boxes represent the CaMV35S minimal promoter and the region encoding its 5′UTR. **(B)** GUS activity was measured in duplicate seed extracts from 10, 17, 11, 11, 11, and 6 independent transgenic lines carrying the -223/+49, -114/-32/-64p35S, -114/+49. -223/-32/-64p35S, -114/-1/35S 5′UTR and -64p35S constructs fused to GUS, respectively. Each symbol represents one transgenic line.

We have previously shown that the region between -223 and +49 (numbers in relation to the transcription start site) is sufficient to confer high expression of the GUS reporter gene in mature embryos (Elorza et al., 2006; **Figure 1**). This region contains three ABRE elements and one RY-like enhancer element, which were shown by mutagenesis to be necessary for high *SDH2.3* promoter activity (Roschzttardtz et al., 2009). In order to evaluate if a promoter fragment comprising the three ABRE and RY elements is sufficient for promoter activity, the region between -114 and -32 was fused to a minimal CaMV35S promoter containing TATA and CAAT boxes (construct -114/-32/-64p35S in **Figure 1A**). Extracts from mature T2 seeds were used to measure GUS activity: a drastic reduction was found when compared to the -223/+49 control construct, showing that the three ABRE and RY elements are necessary but not sufficient to drive high seed expression.

We interpreted that either the region upstream of the ABRE3 element (-223 to -114), the region downstream of the RY element (-32 to +49), or both are required for transcription in seeds. Thus, two additional constructs were assayed, one in which the region between -223 and -114 was deleted (-114/+49 in **Figure 1A**), and the other in which the -223 to -32 fragment was fused to the CaMV35S minimal promoter (-223/-32/-64p35S in **Figure 1A**). Interestingly, GUS activity was not significantly affected by deletion of the promoter to -114 (-114/+49 in **Figure 1B**), indicating that all the elements necessary for high seed expression are present in the -114 to +49 region. In contrast, GUS activity was abolished almost completely when the region downstream the RY element was absent (-223/-32/-64p35S in **Figure 1B**). Furthermore, very weak or no GUS activity was detected when the -114 to -1 region of the *SDH2.3* promoter was fused to the 5′ UTR of the CaMV35S promoter in pBI121 (-114/-1/35S 5′UTR in **Figure 1A**) (**Figure 1B**). Altogether, these results showed that *cis*-elements located between -32 and +49 are necessary for a functional *SDH2.3* promoter, in addition to the upstream ABRE and RY elements. Moreover, they suggest that at least some of these *cis* elements are located in the region encoding the 5′ UTR.

*In silico* analysis revealed the presence of two putative *cis*-elements that may be involved in the regulation of seed gene expression (e.g., Marzabal et al., 2008; Agarwal et al., 2011) and are located downstream of the RY element. These elements, an auxin-responsive element (AuxRE) and a DOF transcription factor-binding site (Supplementary Figure S1A) were mutated either alone or in combination, without significant changes in promoter activity (Supplementary Figure S1B). Thus, these elements play a minor role, if any, in *SDH2.3* promoter activity.

### bZIP Transcription Factors and ABA Regulate *SDH2.3* Expression

**FIGURE 2:**
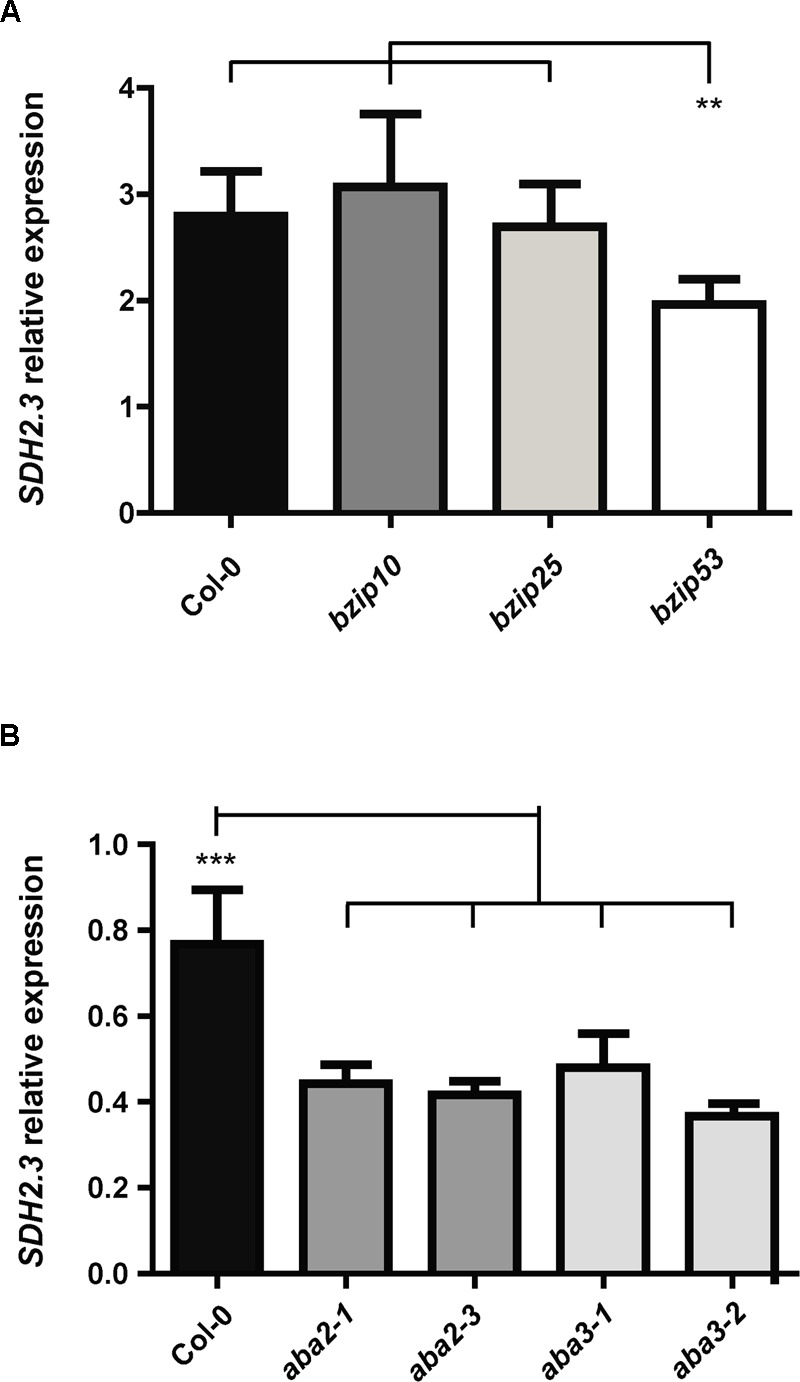
***SDH2.3* expression is reduced in *bzip53* and ABA deficient mutant seeds.** *SDH2.3* transcript levels were determined by qRT-PCR and normalized to clathrin mRNA levels for each sample. **(A)** Expression in dry seeds. Values represent means ± SD of three biological replicates (two technical replicates each). Two asterisks indicate a value for *bzip53* seeds that was determined by the *t*-test to be significantly different from the other three genotypes (*p* < 0.01). **(B)** Expression in *aba* mutant seeds. The last two steps of the ABA biosynthetic pathway are impaired in *aba2* and *aba3* mutants, respectively. Values represent means ± SD of three biological replicates (two technical replicates each). Three asterisks indicate a value in Col-0 seeds that was determined by the *t*-test to be significantly different from the four *aba* mutants at *p* < 0.001.

We have previously shown that transcription factors of the B3 domain family regulate *SDH2.3* expression *in vivo* (Roschzttardtz et al., 2009). In contrast, although we were able to demonstrate that the transcription factors bZIP53, bZIP10, and bZIP25 bind *in vitro* to the ABRE motifs in the *SDH2.3* promoter, no functional data about the *in vivo* role in regulating *SDH2.3* expression was obtained. Accordingly, we first analyzed *SDH2.3* expression in *bzip53, bzip10*, and *bzip25* homozygous mutant seeds (**Figure 2A**). We have chosen the group S1 bZIP53 (Jakoby et al., 2002) because this transcription factor has an important role in seed maturation gene expression (Alonso et al., 2009), and bZIP10 and bZIP25, two members of group C of bZIPs, because they are dimerizing partners of bZIP53 (Ehlert et al., 2006; Weltmeier et al., 2006) and have been shown to induce SSP gene expression (Lara et al., 2003; Alonso et al., 2009).

The accumulation of *SDH2.3* transcripts was significantly reduced in *bzip53* knockdown seeds whereas *bzip10* and *bzip25* mutant seeds did not show any significant difference when compared to wild type seeds (**Figure 2A**). These results reveal that bZIP53 is likely involved in the *in vivo* regulation of *SDH2.3* expression. A plausible explanation for unaltered transcript levels in *bzip10* and *bzip25* mutant seeds may be redundancy between these transcription factors and/or with other bZIPs from the C group.

**FIGURE 3:**
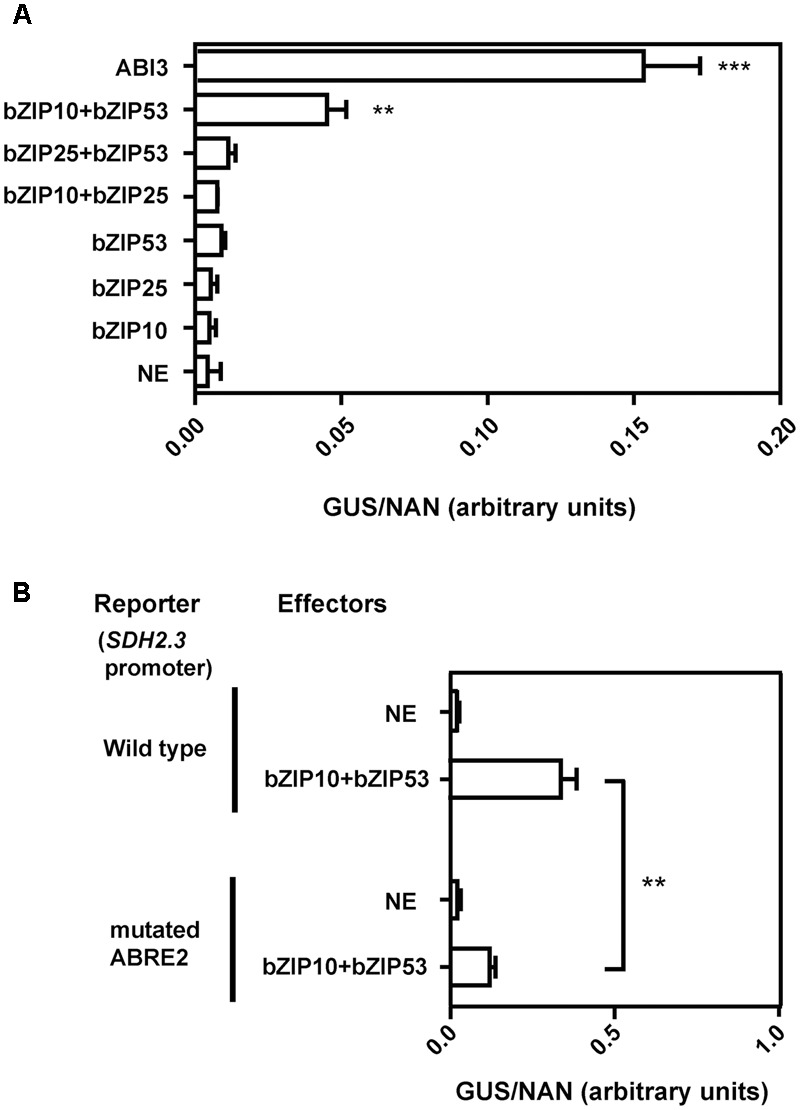
**bZIP53 and bZIP10 transcription factors activate the *SDH2.3* promoter. (A)** *Arabidopsis* protoplasts were transfected with a reporter construct (*SDH2.3* promoter fused to GUS) and effector constructs for expression of either bZIP or ABI3 transcription factors under the control of the CaMV35S promoter. A control plasmid expressing NAN was included in all transfections to normalize GUS activities for differences in transfection efficiencies. x-axis values are given as GUS activity to NAN activity (arbitrary units). NE is no effector(s) (replaced by the pUCpSS vector). Values are means ± SD of three replicates. Asterisks indicate values that were determined by the *t*-test to be significantly different from that in NE (^∗∗^*p* < 0.01; ^∗∗∗^*p* < 0.001). All other GUS/NAN values are not significantly different form that in NE. **(B)** *Arabidopsis* protoplasts were transfected with either the same reporter as in **(A)** (wild type *SDH2.3* promoter fused to GUS) or a reporter construct with a mutated version of the *SDH2.3* promoter, in which a modified ABRE2 element was fused to GUS. Effector constructs expressed bZIP transcription factors. Values are means ± SD of three replicates. Promoter activities in the presence of bZIP53+bZIP10 are significantly different from activities in NE (*p* < 0.001, asterisks not shown). Two asterisks indicate a significant difference at *p* < 0.01.

In a second approach, we performed transient expression analysis in leaf protoplasts using the *SDH2.3* promoter (-114 to +49) to drive GUS expression. This reporter plasmid was cotransfected with effector plasmids expressing bZIP and/or the B3 domain transcription factor ABI3, and a plasmid expressing NAN to normalize for transfection efficiency. Individually bZIP10, bZIP25, and bZIP53 were unable to activate the *SDH2.3* promoter (**Figure 3A**). Only when bZIP53 and bZIP10 were cotransfected a significant increase in promoter activity was observed, showing for the first time that bZIP transcription factors directly activate the *SDH2.3* promoter. When a modified version of the *SDH2.3* promoter was used in which the ABRE2 element was mutated to a sequence that results in a drastic reduction of *in planta* promoter activity (Roschzttardtz et al., 2009), the activation mediated by bZIP53+bZIP10 was significantly reduced, indicating that the regulation of the *SDH2.3* promoter by these bZIP factors requires intact ABRE motifs (**Figure 3B**). In our transient assays ABI3 was able to activate the *SDH2.3* promoter even more efficiently than bZIP53+bZIP10 (**Figure 3A**), and the inclusion of bZIP transcription factors did not produce any additional increase in promoter activation (Supplementary Figure S2). These results confirm our previous data suggesting a direct effect of ABI3 on the *SDH2.3* promoter.

ABRE elements are bound by bZIP transcription factors and are supposed to mediate ABA effects on transcription. To evaluate if ABA is indeed involved in the regulation of *SDH2.3* expression in seeds, i.e., where *SDH2.3* is expressed *in vivo, SDH2.3* transcript levels were measured in wild type and ABA deficient seeds (**Figure 2B**). The level of *SDH2.3* mRNA was significantly lower in seeds from mutants impaired in the last two steps of the ABA synthesis pathway, revealing that ABA is involved in the *in vivo* regulation of seed *SDH2.3* expression.

Altogether, our results show that a -114 to +49 promoter region is necessary and sufficient for seed specific expression of the *SDH2.3* gene, encoding an iron–sulfur subunit of mitochondrial complex II. ABI3, bZIP10/bZIP53 heterodimers, and the hormone ABA regulate *SDH2.3* expression.

### A Complex II Containing SDH2.3 as Iron–Sulfur Subunit Is Present in Mature Embryos Before Imbibition

**FIGURE 4:**
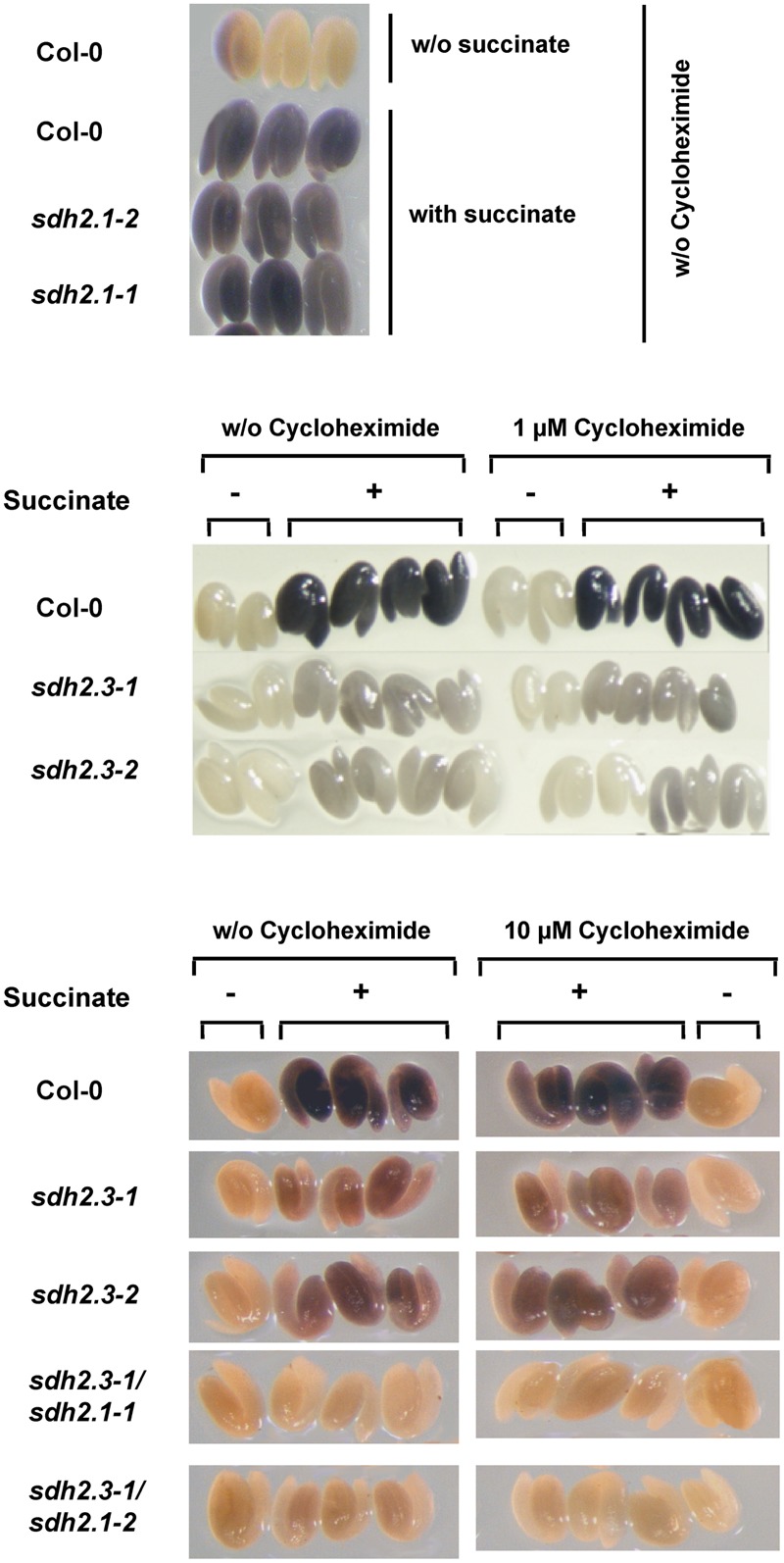
**SDH2.3 containing respiratory Complex II is present in mature embryos before imbibition.** SDH activity was measured in mature embryos from wild type, *sdh2.3* single mutants, *sdh2.3/sdh2.1* double mutants and *sdh2.1* single mutants. Seeds were imbibed in the absence or presence of cycloheximide at the indicated concentrations and subsequent steps were all performed in the same concentration of this protein synthesis inhibitor. As controls of SDH activity embryos were incubated in the same buffer without succinate.

The *SDH2.3* transcript level is high in dry seeds (Elorza et al., 2006). To evaluate if, before imbibition and germination, the SDH2.3 polypeptide is translated and already present in mature embryos and SDH is active, wild type seeds and seeds from single *sdh2.3* mutants, single *sdh2.1* mutants and double *sdh2.3/sdh2.1* mutants were imbibed in the presence (or absence) of the protein synthesis inhibitor cycloheximide. Then all steps, i.e., embryo excision and staining for SDH activity, were performed in the presence (or absence) of cycloheximide. SDH activity was clearly detected in wild type embryos and, most importantly, is insensitive to 1 or 10 μM cycloheximide (**Figure 4**, middle and lower panels). Evidence for effective entry of cycloheximide into the embryo is given by complete inhibition of germination (Supplementary Figure S3). SDH activity was apparently unaffected in *sdh2.1* single mutants (**Figure 4**, upper panel) and was greatly reduced in both *sdh2.3* knockout mutants (**Figure 4**, middle panel). Thus, respiratory Complex II is already present in mature seeds before imbibition and contains mainly SDH2.3 as iron–sulfur subunit. However, SDH activity was detected in *sdh2.3* single mutants, and this activity results from basal expression of *SDH2.1*, since double *sdh2.3/sdh2.1* mutants lack any visible staining (**Figure 4**, lower panel). Semi-quantitative estimation of staining performed as described by Baud and Graham (2006) suggests that single *sdh2.3* mutants have 5–10% of the wild type SDH activity, and that double *sdh2.3/sdh2.1* show negligible activity.

### Complex II Plays a Role in Germination and is Essential for Seedling Establishment

**FIGURE 5:**
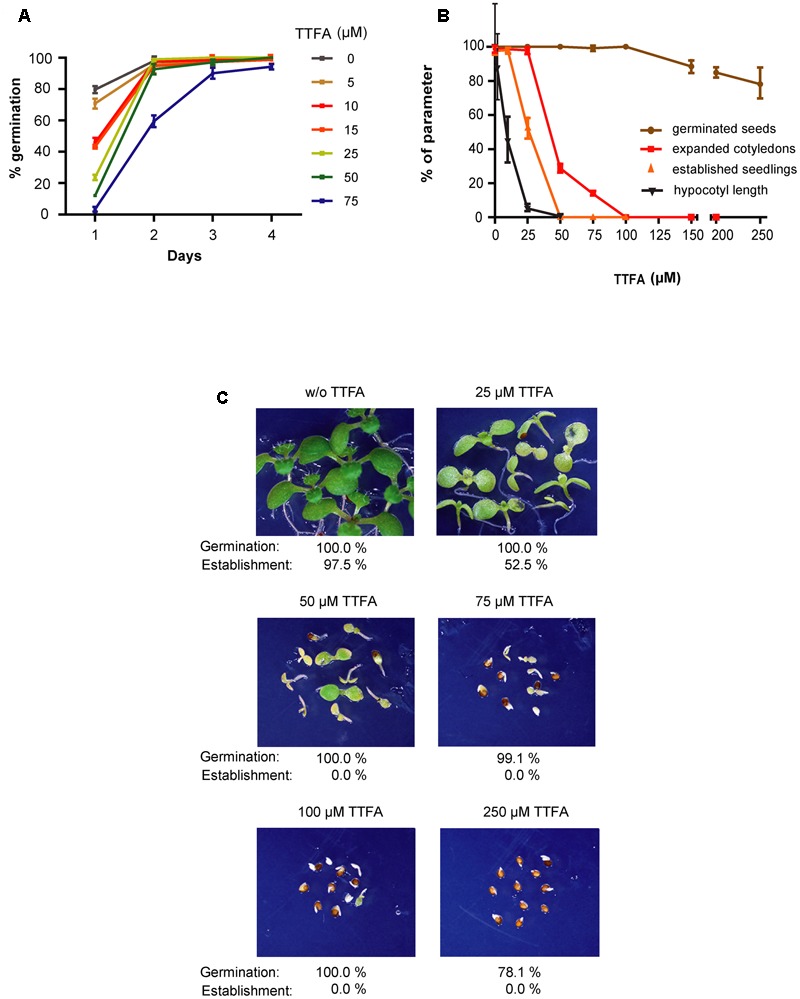
**(A)** Germination is retarded by low TTFA concentrations. Wild type seeds were sown on half-strength MS agar plates without TTFA or with varying concentrations of this complex II inhibitor. Germination was scored at different times and is complete at 4 days. Values are means ± SD of three replicates, each containing 80–200 seeds. **(B,C)** Post-germinative growth is strongly inhibited by TTFA. Seeds were sown on half-strength MS agar plates, stratified, and grown for 10 days under long-day conditions to evaluate germination (brown symbols), cotyledon expansion (red symbols) and seedling establishment (orange symbols). In a different experiment, they were grown in the dark to measure hypocotyl elongation (black symbols). Values are means ± SD of three replicates each containing around 50 sown seeds. Germinated seeds, seedlings with expanded cotyledons and seedlings with true leaves were counted, and values are given as percentages of sown seeds. Hypocotyl lengths are given as percentages of hypocotyl length in the absence of TTFA. **(C)** Photographs were taken at the same magnification after 10 days of growth under long-day conditions.

Germination *sensu-stricto* (radicle protrusion) of *sdh2.3* mutant seeds were delayed when compared to that of wild type seeds, however, all seeds germinated and seedlings established (Roschzttardtz et al., 2009). To further analyze the role of Complex II during germination and early post-germinative growth, low concentrations of TTFA, a known specific non-competitive Complex II inhibitor binding at the ubiquinone pocket (Sun et al., 2005), were shown to be able to delay germination without blocking it (**Figure 5A**). When germination was evaluated at 10 days it was only slightly inhibited by TTFA (**Figure 5B**), and at concentrations higher than 100 μM (e.g., 0.25 mM TTFA inhibits germination by only 22%). Thus, a SDH2.3 containing Complex II appears to play an important but not essential role at very early stages of plant development. By comparison, germination is more sensitive to sodium azide, a cytochrome c oxidase (complex IV) inhibitor, being completely blocked at 0.1 mM (Supplementary Figure S4).

**FIGURE 6:**
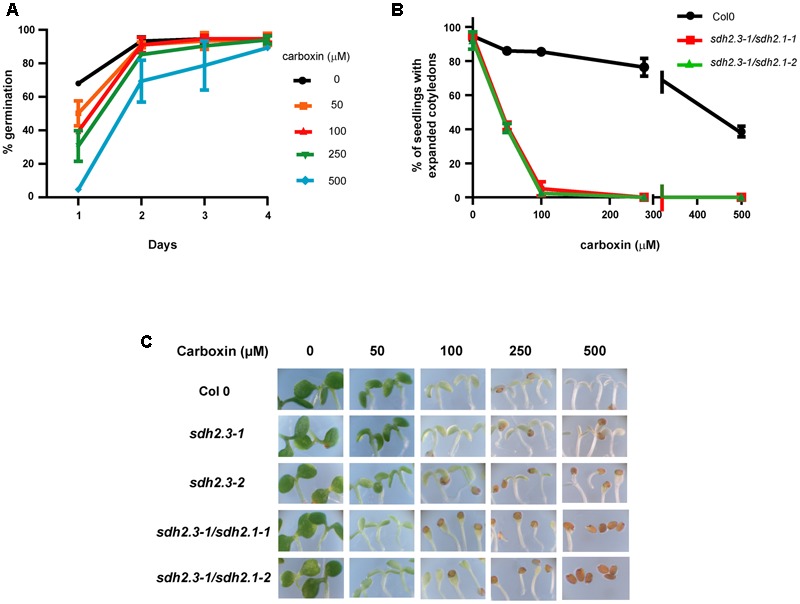
**(A)** Germination is retarded by carboxin. Wild type seeds were sown on half-strength MS agar plates without carboxin or with varying concentrations of this complex II inhibitor. Germination was scored at different times. Values are means ± SD of three replicates, each containing 50–90 seeds. **(B,C)** Post-germinative growth of double *sdh2.3/sdh2.1* mutants is more sensitive to carboxin inhibition. Seeds were sown on half-strength MS agar plates supplemented with different concentrations of carboxin, stratified, and grown under long-day conditions. **(B)** Cotyledon expansion was recorded at 5 days and values are means ± SD (each replicate containing 40–90 seeds). **(C)** Photographs were taken after 8 days of growth.

**FIGURE 7:**
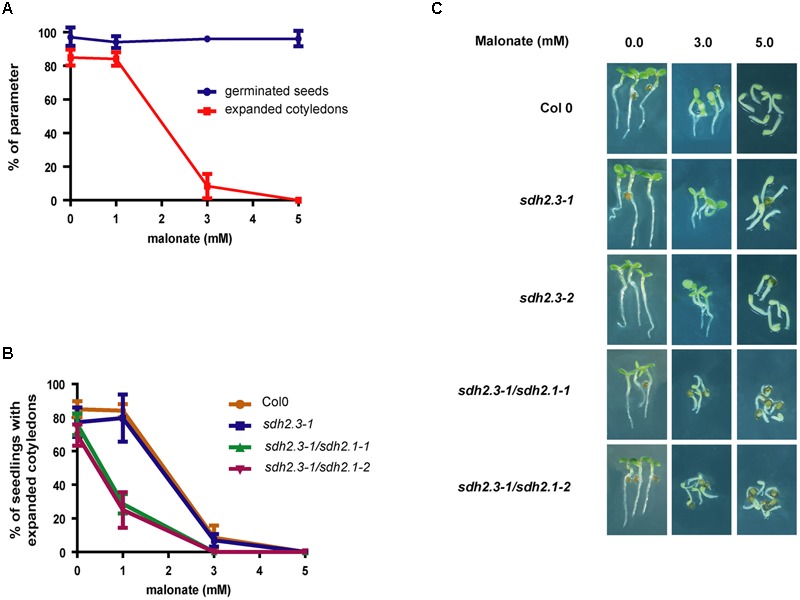
**(A)** Post-germinative growth is inhibited by malonate. Wild type seeds were sown on half-strength MS agar plates without malonate or with varying concentrations of this complex II inhibitor, stratified and grown under long-day conditions. Germination (blue symbols) and cotyledon expansion (red symbols) were evaluated at 30 h and 3 days, respectively. Values are means ± SD of three replicates, each containing 30–50 seeds. **(B,C)** Post-germinative growth of double *sdh2.3/sdh2.1* mutants are more sensitive to malonate inhibition. Seeds were sown, stratified, and grown under long-day conditions. **(B)** Cotyledon expansion was evaluated for each genotype at 3 days, and values are means ± SD of three replicates, each containing 30–60 seeds. **(C)** Photographs were taken after 3 days of growth.

In contrast to the moderate effect of TTFA on germination, cotyledon expansion (and greening), hypocotyl elongation and seedling establishment are more sensitive to TTFA (**Figure 5**). For instance, seedling establishment is completely blocked by 50–75 μM TTFA (**Figure 5B**) and seedlings remain white (**Figure 5C**). Similar results were obtained with two additional Complex II inhibitors: carboxin (**Figure 6**), a non-competitive inhibitor binding at the quinone site, and malonate (**Figure 7**), a competitive inhibitor at the succinate binding site. Importantly, double *sdh2.3/sdh2.1* mutants are more sensitive to these inhibitors (**Figures 6**, **7**), strongly suggesting that seedling greening and establishment is blocked through Complex II inhibition. Altogether, these results suggest that Complex II is essential for chloroplast biogenesis, the acquisition of photosynthetic competence and ultimately seedling establishment.

## Discussion

### Regulation of *SDH2.3* Expression

The *SDH2.3* gene, encoding an isoform of the Complex II iron–sulfur subunit, is the only described TCA cycle and ETC gene to be specifically expressed in the embryo during seed maturation, and this expression is controlled at transcription (Elorza et al., 2006; Roschzttardtz et al., 2009). We have now defined the region (-114 to +49) that contains all the *cis*-elements needed for high seed transcription (**Figure 1**). This region includes between -114 and -32 three ABRE elements and one RY element which are necessary but not sufficient for seed expression. Thus, further elements located between –31 and +49 are required, and at least some of them appear to be present in the region encoding the 5′ UTR since exchanging the *SDH2.3* 5′ UTR for a known functional 5′ UTR inactivates the promoter (**Figure 1**, construct -114/-1/35S 5′ UTR). It is likely that this region (-31 to +49) contains the core promoter elements needed to assemble the transcription pre-initiation complex. Unfortunately, these elements are not well-characterized in plants, particularly in promoters such as the *SDH2.3* promoter that lack a TATA box (e.g., Molina and Grotewold, 2005; Morton et al., 2014).

Group S1 bZIP53 has an important role in seed maturation gene expression (Alonso et al., 2009), and bZIP10 and bZIP25 have been shown to activate SSP gene transcription synergistically with ABI3 (Lara et al., 2003). Here we demonstrate that bZIP transcription factors activate the *SDH2.3* promoter and regulate *SDH2.3* expression *in vivo*. First, we found that *SDH2.3* mRNA levels are reduced in a mutant with lowered *bZIP53* expression (*bzip53* knockdown mutant, **Figure 2A**). And second, bZIP53+bZIP10 activate the *SDH2.3* promoter in leaf protoplasts, and this activation is reduced when the ABRE2 *cis*-element is mutated to prevent transcription factor binding (**Figure 3**). In these transient assays, individual bZIP transcription factors and, interestingly, the bZIP53+bZIP25 combination were unable to activate the *SDH2.3* promoter. This apparent specificity for bZIP10 may be related to the fact that bZIP53/bZIP10 heterodimers bind better *in vitro* to the promoter than bZIP53/bZIP25 (Roschzttardtz et al., 2009). However, we cannot exclude a role for bZIP25 in *SDH2.3* regulation during embryo maturation since it is closely related to bZIP10, they are the group C bZIPs with higher expression in developing seeds (Lara et al., 2003), and non-seed models such as mesophyll protoplasts might not reflect the native situation in seeds. Given that bZIP53 has been identified as the major group S1 bZIP member expressed during seed maturation (Alonso et al., 2009), our results provide strong evidence that the *SDH2.3* gene is a direct target of bZIP53 transcription factor, likely as bZIP53/bZIP10 heterodimers. In addition to its role in the regulation of a respiratory complex subunit (this work) and SSP genes during seed maturation (Alonso et al., 2009), bZIP53 is involved in the regulation of amino acid metabolism during low energy and salt/osmolarity stresses in adult plants (Weltmeier et al., 2006; Dietrich et al., 2011; Hartmann et al., 2015). The results from transient expression assays also confirm our previous data on the direct role of ABI3 in the regulation of *SDH2.3* transcription (**Figure 3A**).

Finally, we have demonstrated that the hormone ABA, known for its role in seed maturation and acquisition of desiccation tolerance, is involved in the regulation of *SDH2.3* expression in seeds (**Figure 2B**). Nevertheless, *SDH2.3* expression is not induced in vegetative tissues by either ABA or saline and osmotic stresses (results not shown). Similar results have been reported for SSP genes and are likely due to the lack of the seed-specific B3 domain transcription factors. Regulation of the mitochondrial ETC by ABA is not without precedent, although only in vegetative tissue. Indeed, expression of the gene encoding Alternative oxidase1a is repressed by ABI4 (ABA-insensitive 4) transcription factor and induced by ABA (Giraud et al., 2009), and ABA reduced the expression of a mitochondrial DEXH box RNA helicase involved in splicing of transcripts encoding complex I subunits (He et al., 2012).

In summary, the *SDH2.3* promoter appears to be regulated in a similar way to that of SSP genes (e.g., 2S albumins): they are induced early in maturation, they have similar *cis*-elements, they are the targets of bZIP and B3 domain transcription factors. However, some differences exist: whereas expression of SSP genes decreases during desiccation, *SDH2.3* mRNA remains high. These differences may be due to different promoter architectures and their interaction with transcription factors.

### Complex II Role during Germination and Establishment

In our previous work, we have shown that *SDH2.3* transcripts accumulate in maturing and dry embryos (Elorza et al., 2006). Here we conclusively demonstrated, by using a protein synthesis inhibitor during imbibition and SDH activity assays, that a SDH2.3 containing Complex II is already present in the embryo, before germination (**Figure 4**). Furthermore, the use of single *sdh2.3* and *sdh2.1* mutants, and double *sdh2.3/sdh2.1* mutants allowed us to conclude that most Complex II (∼ 90%) in mature embryos contains SDH2.3 as the iron–sulfur subunit, a little fraction (∼ 10%) contains SDH2.1, and SDH2.2 is absent. These results are consistent with the previously reported weak expression of a fusion between the *SDH2.1* promoter to GUS in maturing embryos, and the lack of GUS expression with a similar construct for the *SDH2.2* gene (Roschzttardtz et al., 2009).

Germination *sensu-stricto* (until radicle emergence) is shortly followed by seed reserve mobilization which fuel growth until the seedling becomes photoautotrophic, both steps being crucial for seedling establishment (Bewley, 1997; Eastmond and Graham, 2001). In different plant species, including Arabidopsis, seed metabolic activity resumes within minutes of imbibition, and respiration through the standard ETC rapidly increases at the earliest stages of germination (e.g., Ehrenshaft and Brambl, 1990; Benamar et al., 2003; Howell et al., 2006; Sew et al., 2013). Moreover, germination of maize (Ehrenshaft and Brambl, 1990), pea (Benamar et al., 2003), and *Arabidopsis* (Supplementary Figure S4) seeds is dependent on a functional ETC, and mitochondria in dry seeds from maize, rice, and pea rapidly become active during imbibition, relying on succinate and external NADH as substrates (Ehrenshaft and Brambl, 1990; Logan et al., 2001; Benamar et al., 2003; Howell et al., 2006). In sunflower, even mitochondria extracted from dry seeds are capable of succinate-dependent O_2_ consumption and ATP synthesis (Attuci et al., 1991).

These observations are consistent with an active SDH2.3 containing complex II already present in mature *Arabidopsis* embryos (**Figure 4**) and suggest a role for this complex during germination *sensu-stricto*. This role is further supported by the delay in germination observed at low TTFA concentrations (**Figure 5A**) and in mutants lacking SDH2.3 (Roschzttardtz et al., 2009). However, a fully active complex II, although contributing to an efficient germination, is not essential at this early step, and this may be explained by mitochondrial oxidation of other substrates, in particular external NADH as mentioned above. The resumption of respiratory activity depending upon a respiratory system present in the dry embryo and able to oxidize succinate and external NADH and to synthesize ATP very early after imbibition, will be a prerequisite for later mitochondrial biogenesis involving gene expression and import of ETC and TCA cycle components, among others (Logan et al., 2001; Howell et al., 2006; Narsai et al., 2011; Weitbrecht et al., 2011).

At present, we can only speculate about the selective advantage that SDH2.3 may confer to complex II. It is interesting to note that transcripts from all other SDH canonical subunits are present in dry seeds and their levels do not change significantly until 24 h post-imbibition (Supplementary Table S1 in Weitbrecht et al., 2011). A *SDH2.1/SDH2.2* like gene has been described in maize, rice and wheat (Kubo et al., 1999; Sandoval et al., 2004), and in databases we have identified a *SDH2.3* homolog in several monocots and dicots (unpublished results). Thus, the presence of two kinds of SDH2 proteins seems conserved among angiosperms, and a specific embryo iron–sulfur subunit may have evolved to deal with the stressful conditions encountered during desiccation (e.g., to protect iron–sulfur centers from oxidative damage).

Beyond its role in germination, our results indicate that complex II has an essential function during early post-germinative growth, for the acquisition of photosynthetic competence and seedling establishment (**Figures 5**–**7**). Single *sdh2.3* and double *sdh2.3/sdh2.1* mutants are able to switch to photoautotrophism and develop normally in the light (e.g., **Figures 6, 7**), however, interpretation of these results in terms of complex II function is complicated by the fact that *SDH2.2* and *SDH2.1* are rapidly induced after imbibition (3 h), at least at the transcript level (Weitbrecht et al., 2011), and double *sdh2.1/sdh2.2* or triple *sdh2.1/sdh2.2/sdh2.3* are not viable. For these reasons, we use complex II inhibitors and found that they block hypocotyl elongation in the dark and the switch from heterotrophic to autotrophic growth in the light. Interestingly, recent studies have shown that complex I is also required for seedling establishment (Kühn et al., 2015), suggesting a general role for the mitochondrial ETC in providing energy for this crucial developmental step.

After germination, growth and seedling establishment depends largely on fatty acid oxidation. Glyoxysomal β-oxidation converts fatty acids to acetyl-CoA, which is subsequently used to produce succinate via the glyoxylate cycle (Eastmond and Graham, 2001; Graham, 2008). Succinate is then transported to mitochondria, where it can be used as a respiratory substrate or as a gluconeogenic substrate, and in both cases complex II activity would be necessary. Thus, complete complex II inhibition would impair both ATP production and the synthesis of sucrose required to fuel growth of new tissues. Although the precise mechanisms which lead to the failure in chloroplast development are unknown, the link between mitochondrial function and chloroplast development is not without precedent. Indeed, there are a number of non-chromosomal stripe (NCS) mutants in maize that carry deletions in essential mitochondrial genes and are maintained heteroplasmically (e.g., Roussell et al., 1991; Gu et al., 1993). These plants exhibit yellow or pale green stripes on the leaves which result from defective chloroplasts in sectors carrying the mitochondrial mutations, supporting the requirement of mitochondrial function for the development of photosynthetically active chloroplasts.

Few mutants in SDH subunits or assembly factors have been characterized (reviewed in Huang and Millar, 2013). Surprisingly, these studies have revealed complex II roles in stomatal function (Araújo et al., 2011; Fuentes et al., 2011), root elongation (Huang et al., 2013), plant defense to pathogens through ROS generation (Gleason et al., 2011), and pollen and ovule development (León et al., 2007). We have now provided evidence for roles in seed germination and seedling establishment. Future research would be necessary to understand at the tissue-specific level the mechanisms operating to explain this diversity in “the complex roles of this simple enzyme” (Huang and Millar, 2013).

## Author Contributions

FR, JV-C, and XJ design the research. FR, RE-C, and IG carried out the experiments and analyzed the data. FR, RE-C, JV-C, and XJ contributed to writing the manuscript. XJ and JV-C supervised the project.

## Conflict of Interest Statement

The authors declare that the research was conducted in the absence of any commercial or financial relationships that could be construed as a potential conflict of interest.

## Footnotes

1 http://rsb.info.nih.gov/ij/

2 http://www.cambia.org

## Abbreviations

ABA: abscisic acid
ABI3: ABA insensitive 3
ABRE: ABA responsive element
AuxRE: auxin responsive element
bZIP: basic leucine zipper transcription factor
DOF: DNA binding with one zinc finger
ETC: electron transport chain
GUS: β-glucuronidase
NAN: neuraminidase
SDH: succinate dehydrogenase
SSP: seed storage proteins
TCA: tricarboxylic acid
TTFA: thenoyltrifluoroacetone
UTR: untranslated region
