# Supplementary material for: An active Mitochondrial Complex II Present in Mature Seeds Contains an Embryo-Specific Iron–Sulfur Subunit Regulated by ABA and bZIP53 and Is Involved in Germination and Seedling Establishment
Source: Front Plant Sci. 2017 Feb 28;8:277. doi: 10.3389/fpls.2017.00277 (PMC5329045; doi:10.3389/fpls.2017.00277)
Supplement: Supplementary file 1 [file Data_Sheet_1.DOCX]

Supplementary Material

An active mitochondrial complex II present in mature seeds contains an embryo-specific iron-sulfur subunit regulated by ABA and bZIP53 and is involved in germination and seedling establishment

Franko Restovic^1^, Roberto Espinoza-Corral^1^, Isabel Gómez^1^, Jesús Vicente-Carbajosa^2^ and Xavier Jordana^1,^*

*** Correspondence:** Xavier Jordana: [xjordana@bio.puc.cl](mailto:xjordana@bio.puc.cl)

# Supplementary Figures and Tables

## Supplementary Table 1. List of oligonucleotides

| **Name** | **Sequence (5’→ 3’)** | **Description** |
| --- | --- | --- |
| sdh2.3F | TTGGATCCATTGTAACACGTAG | *Bam*HI site underlined |
| sdh2.3R | ATTC**CAT**GGTCTGTTCGCTTGATC | *Nco*I site underlined, start codon in bold |
| mAuxRE-R | GCAGGAGATCTAGATGTGCATG | mutated nucleotides underlined |
| mAuxRE-F | CATGCACATCTAGATCTCCTGC | mutated nucleotides underlined |
| mDOF-F | CCTGCAGATATCTACTCACCAAG | mutated nucleotides underlined |
| mDOF-R | CTTGGTGAGTAGATATCTGCAGG | mutated nucleotides underlined |
| mAuxRE/mDOF-F | CACATCTAGATCTCCTGCAGATATCTACTC | mutated nucleotides underlined |
| mAuxRE/mDOF-R | GAGTAGATATCTGCAGGAGATCTAGATGTG | mutated nucleotides underlined |
| sdh2.3F’ | TTGAATTCATTGTAACACGTAG | *Eco*RI site underlined |
| RYR | GGAGAGAGACATGTGCATGG |  |
| sdh2.3/35S | CACATGTCTCTCTCC*ACAATCCCACTATC* | sequence from the *SDH2-3* promoter underlined, sequence from the CaMV35S minimal promoter in italics. |
| GUS35S-R | ACGTAC**CAT***GGGGGACTGACC* | *Nco*I site underlined, GUS start codon in bold, CAMV35S 5’UTR in italics |
| sdh2.3F2 | CACGTTTGAATTCGACCCGATAC | *Eco*RI site underlined |
| 35S-5UTR1-R | *TAGAGTCCCCCGTGTT*GTCAAAATCCTTGG | sequences complementary to CaMV35S 5’UTR and *SDH2-3* promoter in italics and underlined, respectively |
| 35S-5UTR2-R | GGGGATCCTC*TAGAGTCCCCCG* | overlap with previous primer in italics |
| 35S-5UTR3-R | GACTGACCACCC*GGGGATCCTCT* | overlap with previous primer in italics, overlap with GUS35S-R underlined |
| sdh2.3F3 | AAGCTTCATTGTAACACGTAGTCGCA | *Hind*III site underlined |
| bzip10F | CTCGAG**ATG**AACAGTATCTTCTCC | *Xho*I site underlined, start codon in bold |
| bzip10R | GAATTC**TCA**GTCCACGCATTTTTTCG | *Eco*RI site underlined, stop codon in bold |
| bzip25F | GAGCTC**ATG**CACATCGTCTTCTCTG | *Sac*I site underlined, start codon in bold |
| bzip25R | GGATCC**TTA**ATGCTTGTGATTCC | *Bam*H1 site underlined, stop codon in bold |
| bzip53F | GAGCTC**ATG**GGGTCGTTGCAAAT | *Sac*I site underlined, start codon in bold |
| bzip53R | CCCGGG**TCA**GCAATCAAACATATC | *Sma*I site underlined, stop codon in bold |
| abi3F | GAGCTC**ATG**AAAAGCTTGCATGTGG | *Sac*I site underlined, start codon in bold |
| abi3R | GGATCC**TCA**TTTAACAGTTTGAGAAGTTG | BamHI site underlined, stop codon in bold |
| RTsdh2.3F | TGCATATTGTGCGCGTGTTG |  |
| RTsdh2.3R | TATTCATCGCGGCTATCGCT |  |
| RTclnF | AATACGCGCTGAGTTCCCTT |  |
| RTclnR | AGCACCGGGTTCTAACTCAA |  |
|  |  |  |

## Supplementary Figures


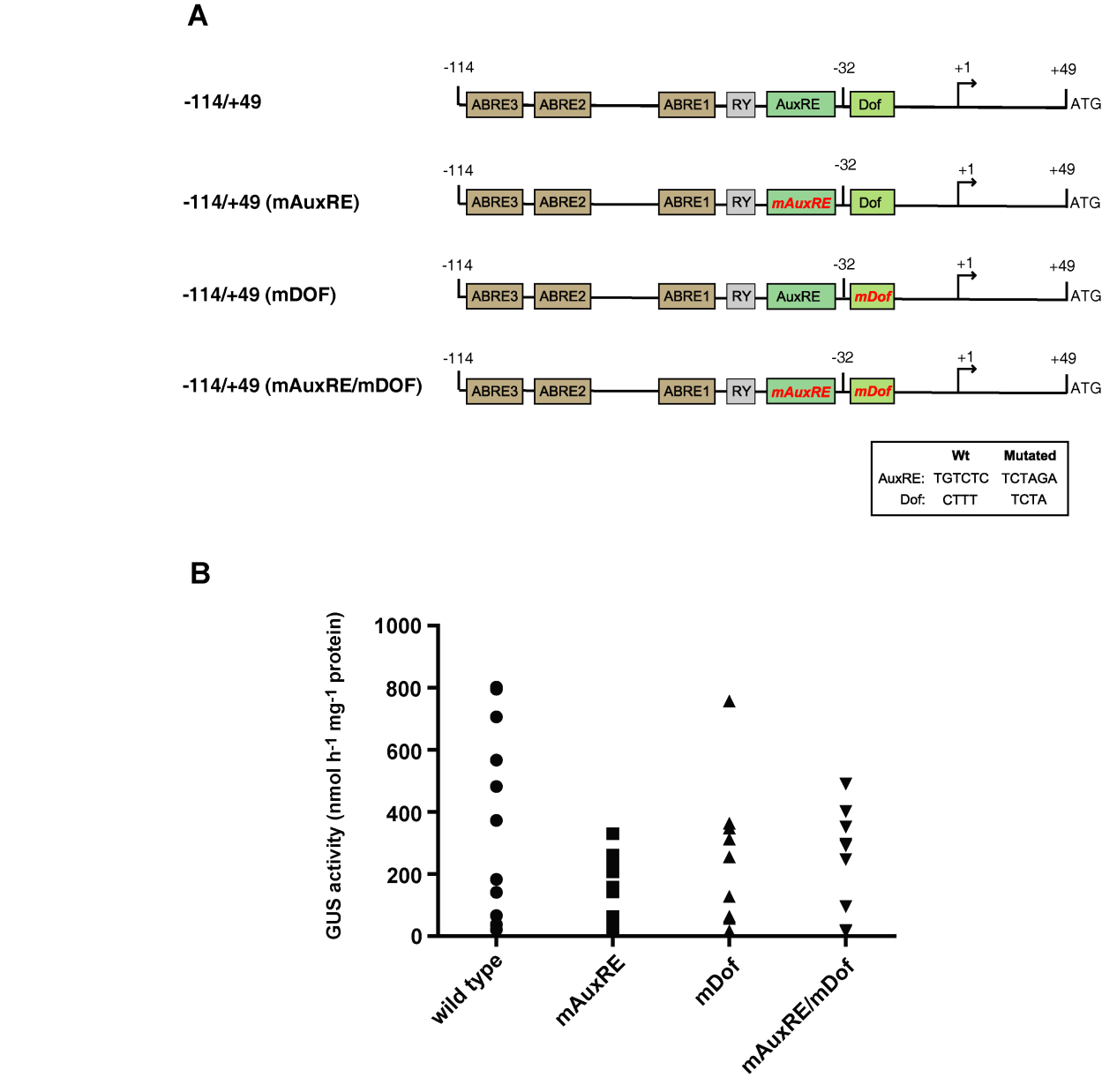


**Supplementary Figure 1.** **Mutation analysis of putative AuxRE and DOF elements in the *SDH2.3* promoter.** **A** Structure of the mutant constructs fused to GUS. Constructs containing substitution mutations in putative AuxRE, DOF site or both were made on the -114/+49 promoter. Mutated elements are indicated as mAuxRE and mDOF, and altered nucleotides are shown below. **B** GUS activity was determined in duplicate T2 seed extracts from 11 (-114/+49 control construct), 11 (mAuxRE), 9 (mDOF) and 9 (mAuxRE/mDOF) independent transgenic lines. Each symbol represents one transgenic line. GUS activity shows means of 379, 178, 256 and 245 nmoles h^-1^ mg^-1^ protein and medians of 373, 207, 255 and 291 nmoles h^-1^ mg^-1^ protein for wild type, mutated AuxRE, mutated DOF, and mutated AuxRE and DOF, respectively. Data analysis using non parametric statistical tests like the Kruskall-Wallis test followed by Dunn’s multiple comparison test or the Mann-Whitney test for pairwise comparisons showed that these differences were not statistically significant.


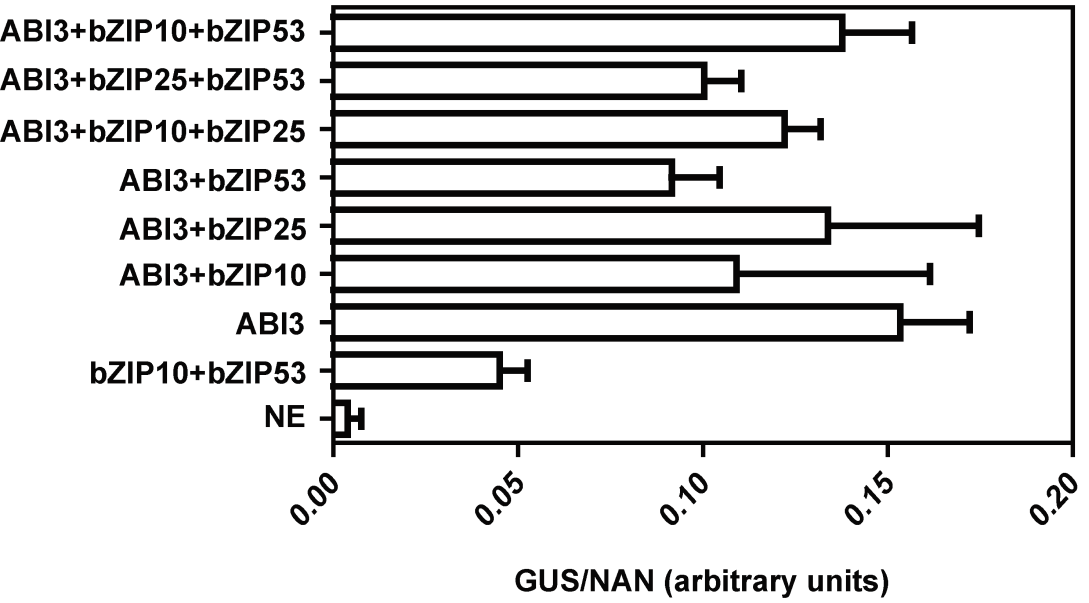


**Supplementary Figure 2. ABI3 activates the *SDH2.3* promoter independently of bZIP transcription factors in transient assays.** Arabidopsis mesophyll protoplasts were transfected with i) a reporter construct containing the *SDH2.3* promoter (-114 to +49) fused to GUS; ii) effector plasmids expressing ABI3 and/or bZIP transcription factors under the control of the CaMV35S promoter; and iii) a control plasmid expressing the neuraminidase gene (NAN) under the control of the CaMV35S promoter. GUS activities were normalized to NAN activities to take account of transfection efficiencies. Values are means ± SD of three replicates. NE is no effector. No significant enhancement of promoter activity was observed when one or two bZIP transcription factors were added to ABI3. All activities are significantly different (p < 0.01 or p < 0.001) from NE activity.


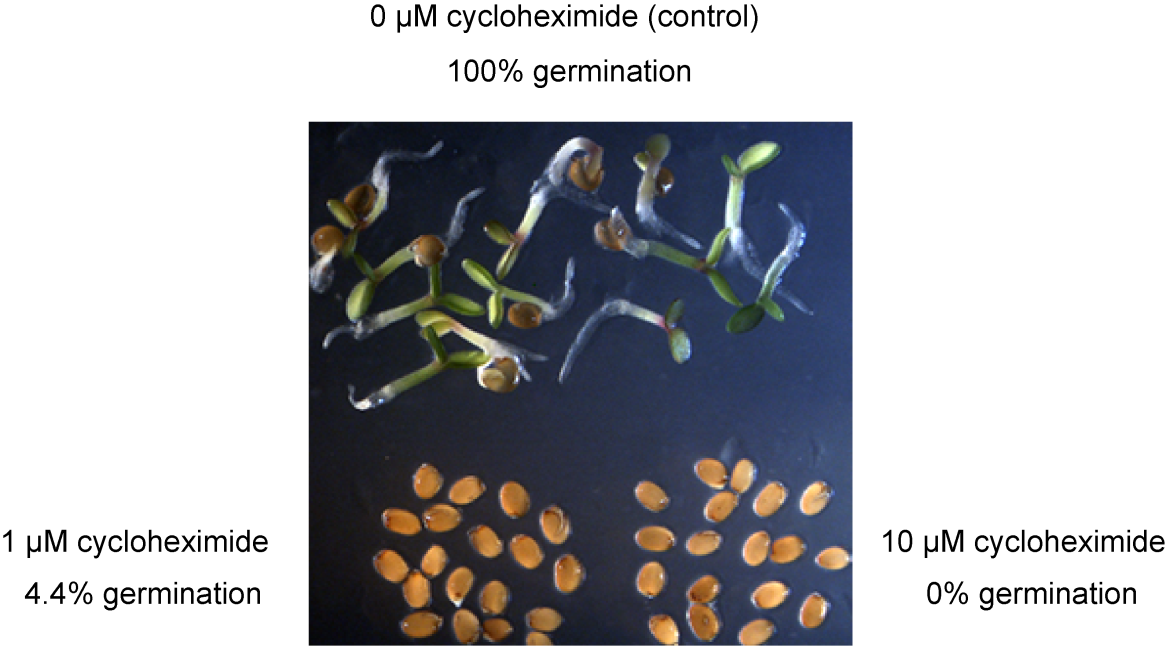


**Supplementary Figure 3. Cycloheximide completely blocks germination.** More than 100 wild type seeds were sown on half-strength MS agar plates without cycloheximide or with 1 and 10 µM of this protein synthesis inhibitor, stratified, and grown under long-day conditions. Germination was scored and photographs taken after three days of growth.





**Supplementary Figure 4. Germination is blocked by sodium azide.** Wild type Col0 seeds (three replicates each containing 40-90 seeds) were sown on half-strength MS agar plates containing varying concentrations of NaN_3_, stratified, and incubated for 7 days under long-day conditions. Values are means ± SD.
